# The effect of psychological interventions on the prevention of chronic pain in adults: a systematic review protocol

**DOI:** 10.1186/s13643-017-0583-7

**Published:** 2017-09-21

**Authors:** Mélanie Bérubé, Céline Gélinas, Manon Choinière, Nancy Feeley, Géraldine Martorella, Stefan Parent, David L. Streiner

**Affiliations:** 1 Centre Intégré Universitaire du Nord de l’Île-de-Montréal (Hôpital du Sacré-Cœur de Montréal) and Ingram School of Nursing, McGill University, 5400 Boulevard Gouin Ouest, Montréal, Québec H4J 1C5 Canada; 2 Ingram School of Nursing, McGill University and Centre for Nursing Research, Jewish General Hospital, 3506 University Street, Montreal, Canada; 3 Department of Anesthesiology, Centre de recherche du Centre of the Centre hospitalier de l’Université de Montréal (CRCHUM), Saint-Antoine Building, 850 Saint-Denis Street, Montréal, H2X 0A9 Canada; 4 College of Nursing, Florida State University, 98 Varsity Way, Tallahassee, FL 32306 USA; 5 Centre de recherche du Centre Hospitalier Universitaire Ste-Justine, 3175 Chemin de la Côte-Ste-Catherine, Montreal, H3T 1C5 Canada; 6 Department of Psychiatry and Behavioural Neurosciences, McMaster University, 100 West 5th Street, Hamilton, Canada

**Keywords:** Psychological risk factors, Acute to chronic pain transition, Secondary prevention, Cognitive-behavioral approach, Adults, Systematic review

## Abstract

**Background:**

Numerous psychological risk and protective factors have been identified as contributing to or preventing the development of the prevalent issue of chronic pain. Systematic reviews of studies on psychological interventions that tackle these factors have shown limited effects on chronic pain. Therefore, implementing psychological interventions before pain becomes chronic has been put forward. However, the efficacy of such interventions in preventing the transition from acute to chronic pain has not yet been systematically assessed.

**Methods:**

The aims of this systematic review are to assess the effects of psychological interventions applied in the acute pain phase on pain severity as well as on physical, psychological, and social functions at 3 months and beyond. Randomized controlled trials including psychological intervention as a treatment of primary interest and participants with pain of less than 3 months duration will be considered. The following comparisons will be undertaken: psychological interventions with (1) standard treatment, (2) information, (3) waiting-list, and (4) active treatment. The primary outcome will be pain severity using indicators such the presence or absence of pain and self-report measures such as the numeric pain intensity rating scale. Secondary outcomes will include pain-related disability, mood, coping with pain, quality of life, health care utilization, and work capability. A systematic review of English and French articles in MEDLINE, Embase, PsycINFO, CINAHL, and the Cochrane Central Register of Controlled Trials will be conducted without date restriction. Extracted data will include demographics and clinical characteristics, sample size, intervention and control group types, assessment tools used, time interval of measurement, fidelity of the intervention, and attrition rate. Standardized mean differences (SMD) and risk ratios with 95% confidence intervals (CI) will be used to assess treatment effects.

**Discussion:**

This systematic review is the first in examining the effects of psychological interventions implemented in the acute pain phase with the objective of preventing chronic pain. Results of this systematic review could provide information on psychological intervention characteristics that are most helpful for individuals with pain and guidance as to when such interventions should be applied in the continuum of care.

**Systematic review registration:**

PROSPERO CRD42016049312

**Electronic supplementary material:**

The online version of this article (10.1186/s13643-017-0583-7) contains supplementary material, which is available to authorized users.

## Background

The acute to chronic pain continuum is commonly described according to a specific timeline. Acute pain is of sudden onset and expected to last less than 3 months [1]. Chronic pain is defined as ongoing or recurrent pain, lasting beyond the usual course of injury healing or more than 3 to 6 months [2]. Chronic pain can negatively affect the person’s physical function [3, 4] and psychological health [5–7]. This condition is also associated with a high socio-economic burden [8, 9].

In a substantive proportion of cases, acute pain will transition to chronic pain. For example, systematic reviews on the epidemiologic literature describing the clinical course of low back pain (LBP) have shown that between 42% and 75% of individuals in industrialized countries that still experience pain 12 months after the first episode [10, 11]. Likewise, reviews of the evidence of post-surgical pain have documented the development of chronic pain in up to 60% of patients depending on the type of surgery [12, 13]. Moreover, a systematic review has described persistent pain in 28% to 93% of patients who suffered limb trauma as long as 7 years post-injury [14]. A recent prospective study has also shown that a third of this population develops moderate to severe neuropathic pain at 4 months post-injury [15].

The development of chronic pain results from complex interactions between biological, psychological, and social factors [12, 16]. There is increasing evidence that the transition from acute to chronic pain is associated with permanent neurophysiological transformations (i.e., central sensitization [17, 18], gliopathy [19], and an emotional shift in the brain circuitry involved in nociception [20]). Moderate to severe acute pain intensity [14, 21], demographic (e.g., female gender and low socio-economic status) [21, 22], as well as surgery (e.g., mastectomy, thoracotomy, and amputation) [12], and injury-related (e.g., lower limb injury and compensable injury) [14, 21] factors have also been shown to play a role in the transition from acute to chronic pain. In addition, several potentially modifiable psychological risk factors for chronic pain have been identified, such as anxiety, depression, pain catastrophizing and pain-related fear [14, 21, 23]. Moreover, psychological variables, such as perceived self-efficacy in pain management [24, 25] and pain acceptance [26–29], have been discussed as potential protective factors.

Systematic reviews of psychological interventions have been conducted in the field of neuropathic pain [30] and non-headache chronic pain [31, 32]. The systematic review performed in neuropathic pain [30] revealed limited evidence, which prevented formal conclusions on the effects of psychological interventions. In the context of non-headache chronic pain, psychological interventions delivered face-to-face [32] or over the Internet [31] showed small to moderate effect sizes on pain outcomes (i.e., pain severity, pain interference with activities, disability, mood, and pain catastrophizing) when compared with usual treatment regimens or a waiting list, and only small effect sizes when contrasted with active control (e.g., physiotherapy and attention-control intervention). Furthermore, most of these effects were observed post-treatment and disappeared at follow-up (i.e., 6 to 12 months post-treatment) [32].

In light of the negative consequences of chronic pain, the prevalence of this condition and its refractoriness to treatment, interest has been growing in the application of early interventions to prevent pain chronicity. In this regard, attempts have been made to determine if psychological interventions can prevent chronic pain and improve patients’ physical, psychological, and social functioning by addressing potentially modifiable psychological chronic pain risk and protective factors.

Psychological interventions include strategies that aim to modify psychological processes that may lead to conditions such as chronic pain [33–35]. These interventions are based on theories of human behavior and behavior change [35]. Applied in the acute to chronic pain transition context, psychological interventions belong to the category of secondary health prevention. The purpose of secondary preventive interventions is to address chronic disease risks and to support the development of behaviors that limit the progression of a condition [36, 37].

Although many terms are used to describe chronic pain preventive psychological interventions, including cognitive-behavioral programs, self-care or self-management interventions or risk-targeted interventions, they all espouse cognitive and behavioral strategies principles also frequently applied in the context of chronic pain [34, 35, 38]. Psychological interventions for the prevention of chronic pain involve programmed activities that focus on psychoeducation (i.e., provision of knowledge on pain and how it operates in order to decrease distress and enhance pain management abilities [39]), prevention or alteration of maladaptive thoughts and emotions, and the promotion of behaviors for effective pain management, such as taking analgesics adequately and staying active [40–43]. These interventions are offered by health professionals such as psychologists, nurses, and physiotherapists as an adjunct to standard pain treatments including analgesics and physiotherapy. A training program provided by experts in the field is usually a prerequisite for health professionals to provide chronic pain preventive interventions [40, 41, 43].

As with other interventions whose purpose is to limit the progression of an illness, the mechanisms through which psychological interventions for the prevention of chronic pain operate are based on three factors influencing human behaviors [36]. The first factor refers to physical and psychological capability to perform a behavior including knowledge and skills. The second factor pertains to the motivation or processes that direct behaviors such as goals, thoughts, emotions, and habits. Finally, the third factor relates to conditions that provide the opportunity for individuals to perform expected behaviors, which involve aspects that may influence engagement with an activity, including the physical, social, and cultural environments [36].

Interventions informed by theories or models of human behavior change (e.g., Health Belief Model [44], Theory of Planned Behavior [45], Self-Efficacy theory [46], and Social Cognitive theory [47]), that take into account capacity, motivation, and/or opportunity factors have been shown to be effective in various health promotion and disease prevention domains such as exercise [48, 49], obesity [50], HIV [51], and cancer [52]. Such positive associations can be explained by the proper fit between the theory and the behaviors to be changed, and mostly, with the factors that sustain these behaviors [36, 53, 54]. Psychological interventions for the prevention of chronic pain are still at their preliminary development and testing stage compared to preventive interventions offered in the context of other health priorities. However, considering what is known on the prevention underpinnings of other health conditions, interventions that address common psychological risk and protective factors of chronic pain (i.e., anxiety, pain catastrophizing, pain-related fear, pain self-efficacy, and pain acceptance) may be effective to prevent the transition from acute to chronic pain.

The treatment of chronic pain often requires the delivery of complex multidisciplinary interventions. Despite annual health care costs exceeding those of heart disease, cancer, and diabetes [8], interventions to resolve the issue of chronic pain have been associated with limited success. Applying interventions before the permanent damages associated with chronic pain occur may improve their benefits. Some clinical trials have demonstrated that psychological interventions can prevent the acute to chronic pain transition [40–43]. However, the efficacy of such interventions in preventing chronic pain has not been assessed comprehensively. A systematic review is needed to critically appraise evidence on psychological interventions aimed at preventing chronic pain as well as to guide future research activities on this topic and knowledge translation.

## Objectives

To assess the efficacy of psychological interventions in preventing chronic pain in adults compared with standard treatments, information, waiting list control, and active treatments.

## Methods/design

### Criteria for study inclusion in this review.

This systematic review has been developed according to the Preferred Reporting Items for Systematic Reviews and Meta-Analyses Protocols (PRISMA-P) [55] as per the checklist presented in an additional file (see Additional file 1). A PICOS question (participants, intervention, comparators, outcomes, study design) was developed to identify criteria for study inclusion as follows:

#### Participants

We will include studies involving adult participants (≥ 18 years old) with non-cancer pain of less than 3 months.

#### Interventions

We will focus on studies that include at least one trial arm consisting of psychological intervention to prevent chronic pain and one comparator arm. We define psychological interventions to prevent chronic pain as psychoeducation, cognitive-behavioral interventions, mindfulness-based stress reduction interventions, mindfulness-based cognitive interventions, acceptance and commitment interventions, hypnotherapy, psychological risks targeted interventions, self-care enhancement or self-management interventions not combined with a standardized physiotherapy program. We will only include studies in which trained individuals provided the intervention when delivered face to face or over the phone.

#### Comparators

Comparators will include standard treatment, information provided through leaflet, video or the web, waiting list control or any form of other active treatment (e.g., exercise, physiotherapy, biofeedback, and attention-control intervention). Information interventions will have to focus on providing general advice on how to manage pain. To ensure that the comparator arm is not a psychological intervention, we will exclude studies in which the comparator includes information on the interactions between the biopsychosocial dimensions of pain or psychoeducation. In case of doubt, we will contact corresponding authors to obtain more details on the information package provided to participants.

#### Outcome measures

According to the Initiative on Methods, Measurement, and Pain Assessment in Clinical Trials (IMMPACT), pain severity as well as physical and emotional functioning are core domains in chronic pain and prevention/treatment trials [56, 57]. Moreover, in chronic pain preventive trials, an emphasis should be placed on the presence and intensity of pain [57]. In this regard, beneficial effects could be defined as the absence of pain or pain below a clinically significant or moderate pain intensity level (e.g., average pain intensity 3 on 10 or greater) at 3 months and more post pain onset. We took into account the IMMPACT core domains to determine our primary and secondary outcomes.

#### Primary outcomes

Pain severity, which will be evaluated by determining the presence or absence of pain and pain intensity measured with validated self-rating instruments [e.g., 0–10 Numerical Rating Scale (NRS) or a 0–10/0–100 visual analogue scale (VAS)] [58].

#### Secondary outcomes

Pain-related disability, mood (e.g., anxiety, depression, pain catastrophizing, and pain-related fear), coping with pain (e.g., pain self-efficacy and coping strategies), quality of life, health care, and social impacts (i.e., health care utilization and return to work), adverse events (e.g., stress or anxiety induced by participation to the intervention and worsening of pain caused by intervention activities) and dropouts for any cause including spontaneous recovery of pain.

#### Study design

We will include randomized controlled trials (RCTs) meeting the following criteria:

- Include a psychological intervention as a treatment of primary interest;
- Provide a description of the psychological intervention content and format;
- Published (or electronically pre-published) in a peer-reviewed journal;
- Include a majority of participants (75%) who had pain for less than 3 months;

Search methods for identification of studies.

#### Electronic searches

A systematic review of English and French articles in MEDLINE (via Ovid), Embase (via Ovid), PsycINFO (via Ovid), CINAHL (via EBSCO host), and the Cochrane Central Register of Controlled Trials (CENTRAL) (via the Cochrane Library) will be conducted without date restriction. In order to reduce selection bias for articles available in other languages, the English abstract will be reviewed to determine if the study should be translated and included. We will also check reference lists of reviews and retrieved articles for additional studies, and we will perform citation searches on key articles. Authors will be contacted when necessary for additional information.

Medical subject headings (MeSH) or equivalent and text word terms will be used. The primary author has developed the search strategy with an experienced health librarian. Search terms will include psychotherapy, behavior therapy, cognitive therapy, self-regulation training, psychoeducation, pain, chronic pain, and RCT. Additional file 2 details the search strategy for MEDLINE, which is suitable for other databases.

### Data collection and analysis

#### Selection of studies

Two reviewers (MB and CG) will independently determine eligibility by reading the abstract of each identified study. Reviewers will read these studies independently and select studies meeting inclusion criteria. Disagreements between reviewers will be resolved by consulting a third reviewer, a senior researcher (MC). We will record data related to eligibility assessment on a standardized form in DistillerSR. To ensure the transparent reporting of identified studies, we will include a PRISMA flow chart in the full review [59]. This flow chart will map out the number of RCTs identified, included and excluded, and the reasons for exclusions.

### Data extraction and management

Three review authors (MB, CG, and GM) and one research assistant trained in data extraction will work independently in team of two to extract data using a standardized form in DistillerSR that will be developed according to the Cochrane Consumers and Communication Review Groups’ data extraction template [60]. Data extraction forms will first be pilot tested with 10 articles (5 articles by team) that do not meet inclusion criteria and refined until reaching ≥ 90% inter-rater reliability [61]. We will also assess the level of data extraction consistency between the reviewers for the articles that meet inclusion criteria using Cohen’s kappa [61]. Inconsistency of data extraction will be resolved by discussion between the two reviewers of the same team, and, if consensus is not reached, a third reviewer will be involved (NF).

Extracted data will include information on publication (title, authors, year, and country), registered trial numbers (when available), population (participant socio-demographic and clinical characteristics, initial and final sample size), intervention type and dosage, control group type and dosage, mode of delivery (e.g., phone, face to face, online video, and sms), type of analysis (e.g., intention-to-treat and completers only), outcomes (measures, time interval of measurement, scores, and standard deviations), fidelity of the intervention, dropouts, and adverse events. We will collate multiple reports of the same study, so that each study rather than each report is the unit of interest in the review. Characteristics of the included studies will be collected in sufficient detail to populate a table of “characteristics of included studies” in the full review.

### Quality assessment

Three authors (MB, CG, and GM) and one research assistant will work independently in team of two to assess risk of bias for each study, using a “risk of bias table” in DistillerSR developed according to the criteria outlined in the Cochrane Handbook for Systematic Reviews of Interventions [60, 62]. We will specifically assess selection bias, performance bias, detection bias, reporting bias, and attrition bias. Judgment of whether high, low, or unclear risk of bias will be made. Disagreement in reviewers’ quality assessment will be discussed to reach consensus with consultation of a third reviewer (DS) when necessary.

### Data synthesis

The PRISMA framework will be used for transparent reporting of RCTs [59]. We will provide a narrative review of the findings from the included studies structured and extracted data. We will calculate effects of preventive psychological interventions when compared to each retained comparators at 3 months and at 6 to 12 months and beyond post-onset to determine their sustainability [57]. We plan to use standardized mean differences (SMD) with 95% confidence intervals (CI) to assess treatment effect with regard to pain intensity and other continuous outcomes. We will analyze dichotomously the presence or absence of pain and, where appropriate, clinically significant changes in outcome scores as determined by the IMMPACT [57, 63] or other sources on assessment tools. In this regard, we will calculate risk ratios (RR) with 95% CI.

Whenever possible, we will use results from an intention-to-treat (ITT) analysis. If standard deviations (SDs) are not provided for continuous outcomes, we will calculate them from standard errors or CI [64]. Cases analysis will be used in presence of missing data and the potential impact of the missing data on the results will be considered.

### Assessment of heterogeneity

We plan to assess heterogeneity by calculating *I* ^2^ statistics for all outcome variables. We will only consider studies with *I* ^2^ less than 50% (i.e., low to moderate heterogeneity) to proceed with a meta-analysis [65]. Factors contributing to heterogeneity will be analyzed with a random-effect regression model [66]. We plan, if the number of articles allows to use population (e.g., patients with back pain, whiplash injury or extremity trauma), intervention dosage, and comparator type (i.e., standard treatments, information waiting list control, or any form of active treatment) as covariates into the meta-regression analysis. We will undertake a meta-analysis only if participants, interventions, comparisons, and outcomes can be judged to be sufficiently similar to provide an answer that is clinically meaningful. Moreover, we will include studies with low risk of bias in the meta-analysis. We plan to meta-analyze outcome data with the random effect model since studies will most likely differ with regard to several aspects of their design [61]. We will perform analyses with the Review Manager (RevMan) software.

### Assessment of reporting biases

We will assess publication bias and selective reporting of results using funnel plot analyses of asymmetry supplemented by the Egger’s test [67, 68]. The possible reasons for asymmetry will be discussed.

### Quality of the evidence

Four review authors (MB, CG, GM, and NF) will work independently in team of two to rate the quality of each outcome (i.e., pain severity, pain-related disability, mood, coping with pain, quality of life and health care/social impacts, and adverse events). We will use the Grades of Recommendation, Assessment, Development and Evaluation (GRADE) system to rank the quality of the evidence using the GRADE profiler Guideline Development Tool software [69], and the guidelines provided in Chapter 12.2 of the Cochrane Handbook for Systematic Reviews of Interventions [60].

### Subgroup analysis

We will conduct subgroup analyses according to populations (i.e., patients with back pain, whiplash injury, or extremity trauma), comparator type, as well as intervention dosage, and modes of delivery if sufficient data are available. We will use a random-effect regression model to test for heterogeneity [70, 71].

### Sensitivity analysis

The need for sensitivity analysis will be identified during studies analysis. Considering that outcomes associated with preventive interventions are often evaluated at different time points, we will test the robustness of effects by excluding effect sizes that will be approximated due to missing data [72].

## Discussion

Chronic pain has been associated with permanent neurophysiological transformations, which may contribute to its resistance to treatments [34]. In their report on pain relief, the Institute of Medicine of the National Academies [1] called for pain education on self-management and the role of biological and psychosocial factors early in the process—to prevent pain from becoming chronic. Applying psychological interventions early after pain onset to prevent pain chronicity appears to be an interesting solution. To our knowledge, this systematic review is the first in assessing at the effects of psychological interventions applied in the acute pain phase on chronic pain. The inclusion of published studies only is a limitation of this review. Nonetheless, results could provide guidance on the best timing to offer comprehensive pain management treatments to patients. The findings could also point to psychological intervention components, dosages, and modes of delivery that are associated with the optimal outcomes. Ultimately, this systematic review may enhance access to treatments that aim to limit the disabling issue of chronic pain and associated social cost.

## Additional files

Additional file 1: This file describes where to find essential items of a systematic review. (DOCX 35 kb)

Additional file 2: This file describes the search strategy to identify relevant studies. (DOCX 102 kb)

## Abbreviations

CI: Confidence interval
CINAHL: Cumulative Index to Nursing and Allied Health Literature
GRADE: Grades of Recommendation, Assessment, Development and Evaluation
HIV: The human immunodeficiency virus
IMMPACT: Initiative on Methods, Measurement, and Pain Assessment in Clinical Trials
ITT: Intention-to-treat
MEDLINE: Medical Literature Analysis and Retrieval System Online
MeSH: Medical Subject Headings
NRS: Numerical Rating Scale
PICOS: Participants, interventions, comparisons, outcomes and study design
PRISMA: Preferred Reporting Items for Systematic Reviews and Meta-analysis
PRISMA-P: Preferred Reporting Items for Systematic Reviews and Meta-analysis Protocols
RCT: Randomized controlled trial
SMD: Standardized mean difference
VAS: Visual analog scale
