# Supplementary material for: The effect of psychological interventions on the prevention of chronic pain in adults: a systematic review protocol
Source: Syst Rev. 2017 Sep 21;6:190. doi: 10.1186/s13643-017-0583-7 (PMC5609012; doi:10.1186/s13643-017-0583-7)
Supplement: Supplementary file 2 — This file describes the search strategy to identify relevant studies. (DOCX 102 kb) [file 13643_2017_583_MOESM2_ESM.docx]

Additional file 2. Search strategy in Ovid MEDLINE

| <1946 to December Week 1 2016> | |
| --- | --- |
| # | Search Statement |
| 1 | exp Psychotherapy/ |
| 2 | behavior therapy/ or cognitive therapy/ |
| 3 | exp Biofeedback, Psychology/ |
| 4 | behavio#r therap*.tw. |
| 5 | cognitive therap*.tw. |
| 6 | (relax* adj3 technique*).tw. |
| 7 | (relax* adj3 (technique* or therap*)).tw. |
| 8 | meditat*.tw. |
| 9 | psychotherap*.tw. |
| 10 | (psychological adj2 (treatment* or therap*)).tw. |
| 11 | "group therapy".tw. |
| 12 | "self-regulation training".tw. |
| 13 | "coping skill*".tw. |
| 14 | "pain-related thought*".tw. |
| 15 | (behavio#r* adj3 rehabilitat*).tw. |
| 16 | (psychoeducation adj2 group*).tw. |
| 17 | (psycho-education adj2 group*).tw. |
| 18 | ("mind and body relaxation technique*" or "mind-body relaxation technique*").tw. |
| 19 | exp mind-body therapies/ or relaxation therapy/ |
| 20 | or/1-19 |
| 21 | Chronic Pain/ |
| 22 | pain*.tw. |
| 23 | 21 or 22 |
| 24 | 20 and 23 |
| 25 | adolescent/ or exp child/ or infant/ |
| 26 | 24 not 25 |
| 27 | randomized controlled trial.pt. |
| 28 | controlled clinical trial.pt. |
| 29 | randomized.ab. |
| 30 | placebo.ab. |
| 31 | drug therapy.fs. |
| 32 | randomly.ab. |
| 33 | trial.ab. |
| 34 | groups.ab. |
| 35 | 27 or 28 or 29 or 30 or 31 or 32 or 33 or 34 |
| 36 | exp animals/ not humans.sh. |
| 37 | 35 not 36 |
| 38 | 26 and 37 |
